# Postnatal Cytomegalovirus Exposure in Infants of Antiretroviral-Treated and Untreated HIV-Infected Mothers

**DOI:** 10.1155/2014/989721

**Published:** 2014-03-03

**Authors:** Sarah A. Meyer, Daniel J. Westreich, Emily Patel, Elizabeth P. Ehlinger, Linda Kalilani, Rachel V. Lovingood, Thomas N. Denny, Geeta K. Swamy, Sallie R. Permar

**Affiliations:** ^1^Department of Medicine, Children's Hospital Boston, Boston, MA 02115, USA; ^2^Department of Epidemiology, UNC-Chapel Hill, Chapel Hill, NC 27599, USA; ^3^Department of Obstetrics and Gynecology, Duke University Medical Center, Durham, NC 27710, USA; ^4^Division of Viral Pathogenesis, Beth Israel Deaconess Medical Center, Boston, MA 02115, USA; ^5^College of Medicine, University of Malawi, Blantyre, Malawi; ^6^Human Vaccine Institute, Duke University Medical Center, Durham, NC 27710, USA

## Abstract

HIV-1 and CMV are important pathogens transmitted via breastfeeding. Furthermore, perinatal CMV transmission may impact growth and disease progression in HIV-exposed infants. Although maternal antiretroviral therapy reduces milk HIV-1 RNA load and postnatal transmission, its impact on milk CMV load is unclear. We examined the relationship between milk CMV and HIV-1 load (4–6 weeks postpartum) and the impact of antiretroviral treatment in 69 HIV-infected, lactating Malawian women and assessed the relationship between milk CMV load and postnatal growth in HIV-exposed, breastfed infants through six months of age. Despite an association between milk HIV-1 RNA and CMV DNA load (0.39 log_10_ rise CMV load per log_10_ rise HIV-1 RNA load, 95% CI 0.13–0.66), milk CMV load was similar in antiretroviral-treated and untreated women. Higher milk CMV load was associated with lower length-for-age (−0.53, 95% CI: −0.96, −0.10) and weight-for-age (−0.40, 95% CI: −0.67, −0.13) *Z*-score at six months in exposed, uninfected infants. As the impact of maternal antiretroviral therapy on the magnitude of postnatal CMV exposure may be limited, our findings of an inverse relationship between infant growth and milk CMV load highlight the importance of defining the role of perinatal CMV exposure on growth faltering of HIV-exposed infants.

## 1. Introduction

Despite significant scale-up of antiretroviral prophylaxis, 260,000 pediatric HIV-1 infections are still diagnosed annually [1, 2], of which nearly half are a result of breastfeeding [3]. In the United States, it is recommended that HIV-infected mothers use replacement feeding. However, replacement feeding in resource-poor countries is associated with increased mortality from diarrheal disease, pneumonia, and other infectious diseases [4]. In these regions, the World Health Organization recommends that HIV-infected women breastfeed their infants in the setting of maternal and/or infant antiretroviral prophylaxis, which considerably reduces postnatal HIV-1 transmission [5–7].

Another significant viral pathogen that is vertically transmitted is cytomegalovirus (CMV). Similar to HIV-1, CMV can be transmitted *in utero*, peripartum, or via breastfeeding. Congenital transmission has a well-established association with fetal growth restriction, sensorineural hearing loss, and neurodevelopmental abnormalities [8, 9]. Prior to the advent of highly active antiretroviral therapy (HAART), reported congenital CMV rates were high among HIV-exposed and HIV-infected infants [10–12]. However, studies of congenital CMV in the HAART era show lower rates of congenital CMV in HIV-exposed, uninfected infants [13]. In contrast to congenital CMV transmission, peri- or postpartum CMV transmission is typically asymptomatic in healthy full-term infants. However, peripartum CMV acquisition in HIV-infected infants is an important predictor of morbidity and mortality [14], leading to higher rates of HIV-1 progression. Mounting evidence suggests that growth and development of HIV-exposed, uninfected infants are also adversely affected by perinatal CMV acquisition [15]. Moreover, high rates of symptomatic perinatal CMV infection have been described in HIV-exposed infants, an effect that may be modulated by maternal HAART [16]. Perinatal CMV infection may contribute to the recognized growth impairment of HIV-exposed, uninfected infants. Thus, it is important to establish the pathogenesis of perinatal CMV transmission in HIV-exposed infants and the impact of maternal HAART on infant CMV exposure.

CMV shedding in milk of CMV-seropositive women is common, with postnatal CMV transmission rates up to 70% [17–20]. Breast milk CMV load may be independently associated with the risk of postnatal CMV transmission [21, 22], especially in HIV-1-infected women as a direct correlation between CMV and HIV-1 breast milk viral loads has been described in untreated HIV-infected women [23]. While maternal HAART can effectively reduce HIV-1 RNA load in breast milk, its effect on CMV shedding is not established. Moreover, it is unclear if maternal HAART can reduce perinatal CMV transmission and associated disease. Establishing the impact of maternal HAART on CMV exposure and the effects of CMV exposure on growth and development of HIV-exposed infants is important to improving infant survival in regions of high HIV-1 prevalence. In this study, we aimed to determine the impact of maternal HAART on milk CMV shedding. Further, we evaluated the impact of milk CMV load on the growth of breastfed, HIV-exposed infants.

## 2. Materials and Methods

### 2.1. Participants and Specimens

Sixty-nine HIV-1-infected pregnant women testing positive by rapid antibody test were recruited from two rural health clinics outside Blantyre, Malawi, between 2009 and 2010 and consented for enrollment in this pilot study [24, 25]. The study and informed consent were approved by the College of Medicine Research and Ethics Committee in Malawi and institutional review boards at each participating US institution. Maternal peripheral blood CD4+ T-cell count and plasma HIV-1 RNA viral load were performed during the third trimester and women with confirmed infection were enrolled at delivery. Maternal antiretroviral use was assessed at each follow-up visit (delivery, 4–6 weeks, 3 months, and 6 months); untreated mothers and all infants were administered single dose Nevirapine at delivery. All women were also screened for CMV IgG by ELISA using the third trimester blood sample (*Trinity Biotech*). Breast milk from right and left breasts was collected separately between four to six weeks postpartum. Mothers were counseled to exclusively breastfeed for the first six months and were provided with a peanut-based food supplement for their own nutrition for six months postpartum. Per national guidelines at the time, HIV-infected mothers were counseled at six months postpartum to choose to continue breastfeeding or rapid weaning if replacement feeding was a viable option.

Infants were tested for HIV-1 infection by blood DNA PCR at birth (for *in utero* infection), four to six weeks of age (for peripartum infection), and every three months of life until weaning (for postnatal infection). Positive results were confirmed by repeated blood DNA PCR and plasma RNA load. Infant growth parameters of height (crown-heel length) and weight (by infant scale) were monitored at birth, four to six weeks, three months, and six months. The weight-for-length, length-for-age, and weight-for-age *Z*-scores were determined using the WHO Anthro program (v3.2.2). Congenital CMV infection was diagnosed by cord blood tested for CMV DNA PCR and IgM, as infant urine and saliva were not collected. No infants were positive for CMV IgM (*Trinity Biotech*). Thus, infants with positive CMV DNA PCR of cord blood were considered to be congenitally infected with CMV. All infants included in the growth analysis at six months of age were still breastfeeding.

### 2.2. Laboratory Assays

HIV-1 RNA load was measured using the Roche Cobas Ampliprep/Cobas TaqMan 48 for HIV-1 load assay. Breast milk supernatant was diluted 1 : 5 in phosphate-buffered saline (PBS) prior to analysis, whereas plasma was diluted 1 : 10. The minimum levels of detection for this assay were 480 viral RNA copies/mL of plasma and 240 viral RNA copies/mL of breast milk. The laboratory performing these assays was enrolled in the National Institute of Allergy and Infectious Diseases Division of AIDS Virology Quality Assessment Program and certified for HIV-1 load determinations [25]. Milk CMV DNA load was determined by quantitative PCR as previously described [26]. The limit of quantification was 87 copies/mL. If virus amplification was detected but was below the level of the minimum virus standard, a value of half the minimum of detection was assigned. Sodium and potassium concentration of milk supernatant was measured using the Gen2 Ion Selective Electrode on the Roche Cobias c501 platform (Roche Diagnostics), with a sodium-potassium ratio >1 indicative of subclinical mastitis [27, 28].

### 2.3. Statistical Analysis

Distributions of demographic and laboratory characteristics were described using medians and interquartile ranges; differences between medians were described using two-sided *P* values from Wilcoxon rank sum tests. We estimated the effects of HAART on CMV breast milk load using linear regression (crude and adjusted) for infant birth weight and gestational age and maternal mastitis, age, and CD4 count. Confidence intervals around the CMV and HIV-1 transmission rates were calculated with a continuity correction. For the analysis of viral load data in each breast, we applied a goodness-of-fit analysis (quasi-Akaike's information criterion) to determine whether an independent or exchange structure regression model was a better fit, and the results determined that the independent structure was a better fit. Thus, a generalized estimating equation (GEE) with an independent correlation matrix to control for the within-woman correlation was used for the analysis of both breasts and each breast separately (right or left) from each individual woman. Cross-sectional associations between HIV-1 and CMV loads were described using scatterplots and linear regression to obtain slopes and *R* ^2^ values. Comparisons of the frequency of HIV-1 or CMV transmission between the groups were performed using the chi squared test. Finally, we estimated the effect of milk CMV DNA and HIV-1 RNA load at four to six weeks on infant growth *Z*-scores and the change in *Z*-scores for weight-for-length, length-for-age, and weight-for-age using linear regression both crude and adjusted for infant birth weight and gestational age and maternal CD4 count, age, and HAART use.

## 3. Results

### 3.1. Clinical Characteristics of HIV-Infected, CMV-Seropositive Lactating Women and Their Infants

Sixteen women initiated HAART (Trioimmune: Stavudine, Lamivudine, and Nevirapine) prior to enrollment, with two on therapy prior to pregnancy and 14 initiating HAART during pregnancy. Single dose Nevirapine was provided during labor to the remaining 53 untreated women and all infants. HAART-treated women were significantly older than untreated women. All women were CMV IgG seropositive. HAART-treated women had a similar peripheral CD4+ T-cell count to that of the untreated women during the third trimester (Table 1). Maternal plasma HIV-1 load during the third trimester was significantly lower in HAART-treated versus untreated women with log_10_ plasma HIV-1 RNA load 2.4 versus 4.0, respectively. Breast milk HIV-1 RNA load was detectable at 4–6 weeks postpartum in at least one breast in two of 16 women on HAART treatment (12.5%) versus 33/53 (62.3%) untreated women (*P* = 0.0005 by chi squared test). Thus, the median milk HIV-1 RNA load measured was similar between the two groups, but the range of the milk virus load was significantly higher in the untreated women (Table 1). Despite counseling on benefits of exclusive breastfeeding, of the 67 women who reported the date of initiation of mixed feeding, 10 women reported initiation of mixed feeding prior to 5 months of life (14.9%), 26 women reported initiating mixed feeding in the infant's sixth month of life (38.8%), 24 reported initiating mixed feeding after six months of age (35.8%), and seven women reported not initiating mixed feeding before weaning at six months of life (10.4%). While symptoms or signs of mastitis (such as breast soreness, erythema, or induration) were not reported by any of the subjects, subclinical mastitis was detected at four to six weeks postpartum in a similar proportion of treated and untreated women (Table 1).

*In utero* or peripartum HIV-1 transmission occurred exclusively in the untreated group (13%, 95% CI [0.06, 0.26]), despite the use of single dose Nevirapine. Moreover, postpartum HIV-1 transmission occurred only in the untreated group (6%, 95% CI [0.01, 0.17]). When these modes of HIV-1 transmission were combined, there was a trend towards a higher rate of vertical HIV-1 transmission in the untreated group compared to the treated group (*P* = 0.06 by chi square test). Three infants were found to be congenitally infected with CMV by a positive cord blood CMV PCR: two in the treated and one in the untreated group and none of these overlapped with the HIV-infected infants. Low birth weight (<2500 g) was more prevalent in the HAART-treated (25%) than the untreated (2%) group. Moreover, preterm birth (<37 weeks gestation), assessed by Ballard score [29], trended towards a higher incidence in the HAART-treated versus untreated group (Table 1). There was no association between preterm birth and CMV infection in this cohort (*P* = 0.82 by chi square test), yet with only three congenital CMV infections in this cohort, the power to detect associations is limited.

### 3.2. Effect of Maternal HAART and Subclinical Mastitis on Milk CMV DNA Load

CMV load was quantitated in milk collected from each breast at four to six weeks postpartum. We assessed the viral loads in milk collected from each breast due to known potential discordance of HIV-1 RNA load between breasts and potential for unilateral mastitis [25, 28]. The average CMV load from right and left breasts of HAART-treated women was similar to that of untreated women (Table 1). To further assess the effect of HAART on milk CMV load, we determined the difference in log_10_ copies/mL milk CMV load from both breasts and right and left breasts separately, associated with maternal HAART. There was no association between HAART and the magnitude of milk CMV load (Table 2). When the results were adjusted for maternal age, CD4 count, mastitis, and infant gestational age and birth weight, there was a trend towards a decrease in milk CMV DNA load in both breasts and right and left breasts with maternal HAART (log_10_ difference in milk CMV DNA load: −0.33, −0.21, and −0.50, resp.), though the 95% confidence intervals (CI) included zero in all comparisons.

We assessed the difference in log_10_ copies/mL milk CMV load from both breasts and right and left breasts, associated with subclinical mastitis. In both the raw and adjusted comparison of CMV load detected in milk, there was no association between mastitis and CMV load (log_10_ difference in milk CMV load associated with subclinical mastitis: 0.23, −0.47, and 0.46, for both breasts, left breast, and right breast, resp.). After adjustment for maternal age, HAART use, and CD4 count and infant birth weight and gestational age, the difference in milk CMV DNA load by subclinical mastitis remained nonsignificant. Thus, subclinical mastitis was not associated with elevated milk CMV load in our population.

Finally, we compared the milk CMV DNA load among HIV-transmitting and HIV-nontransmitting mothers, combining all HIV transmission modes in one group due to the small number of total HIV transmissions. Interestingly, the CMV DNA load was higher in transmitting mothers (mean log_10_ milk CMV DNA load = 4.99) compared to nontransmitting mothers (mean log_10_ milk CMV DNA load = 3.81) (*P* = 0.003). However, CMV DNA load in milk of mothers of congenitally CMV-infected infants (mean log_10_ milk CMV DNA load = 3.42) was similar to that of mothers of infants who did not congenitally transmit CMV to their infants (mean log_10_ milk CMV DNA load = 3.99) (*P* = 0.31).

### 3.3. Relationship between HIV-1 RNA and CMV DNA Loads in Breast Milk

**Figure 1:**
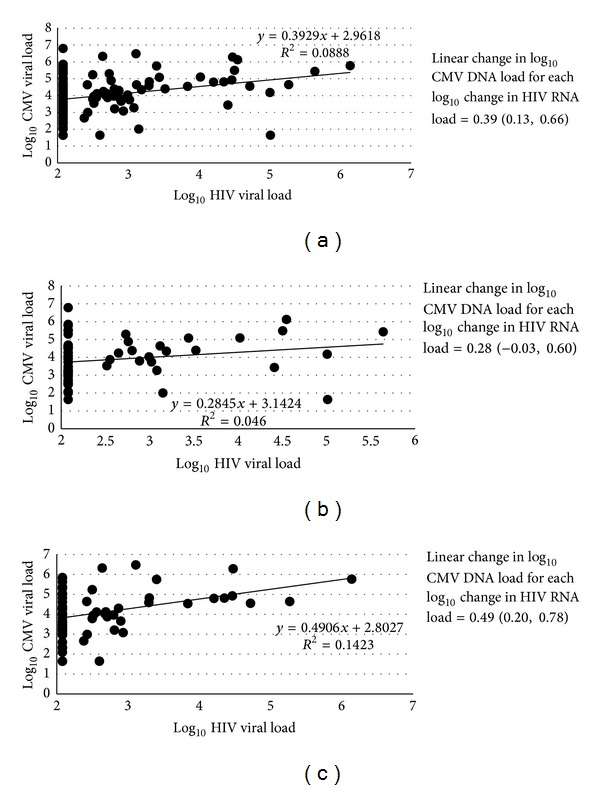
Cross-sectional associations between milk log_10_ HIV-1 RNA load and log_10_ CMV DNA load at four to six weeks postpartum, in both breasts (a), left breast only (b), and right breast only (c). Slope and *R* ^2^ values are indicated on each graph; the difference in log_10_ CMV DNA load for each log_10_ difference in HIV-1 RNA load is presented to the right of each graph.

CMV DNA load in mucosal secretions has previously been directly correlated with the magnitude of the HIV-1 RNA load in the same compartment [23, 30]. Thus, we assessed the cross-sectional relationship between milk HIV-1 RNA and CMV DNA load in our cohort of HAART-treated and untreated HIV-infected women at four to six weeks postpartum. Using a linear regression model, we determined the rise in log_10_ CMV copies/mL associated with a one log_10_ rise in HIV-1 copies/mL (Figure 1). The difference in log_10_ milk CMV DNA load for every one log_10_ rise in milk HIV-1 RNA load was estimated at 0.39 (95% CI: 0.13–0.66) for both breasts, 0.28 for the right breast (95% CI: −0.03–0.60), and 0.49 for the left breast (95% CI: 0.20–0.78). However, there was no direct correlation between the milk viral loads in this cohort (correlation coefficient = 0.298, 0.214, and 0.377 for both, left, and right breasts). Thus, there was a weak association between the magnitude of the milk HIV-1 RNA and CMV DNA load in this cohort of HIV-infected, lactating women.

### 3.4. Association between Maternal Milk CMV DNA Load and Postnatal Growth of HIV-Exposed, Breastfed Infants

We evaluated the association between the magnitude of postnatal CMV exposure in HIV-exposed, uninfected infants and postnatal growth. Three of the HIV-exposed infants had a positive cord blood CMV DNA PCR, for a congenital CMV transmission rate of 4%, similar to previous reports for HIV-exposed infants [13, 31]. For this analysis of postnatal CMV exposure and infant growth, we removed infants that were congenitally or perinatally infected with HIV-1 (*n* = 10) and congenitally infected with CMV (*n* = 3). One uninfected infant died prior to 6 months of age (*n* = 1); thus the analysis was performed on a total of 55 infants. Infant plasma was not available for further CMV testing after birth to determine the incidence of postnatal CMV acquisition. As described in Table 3, there was a significant reduction in crude and adjusted length-for-age *Z*-score and the weight-for-age *Z*-score at six months of age per log_10_ increase in milk CMV DNA load, with adjusted analysis controlling for birth weight and gestational age and maternal CD4 count, age, and HAART use. As exposure to CMV via breastfeeding is a postnatal exposure, we next assessed the change in the *Z*-score of growth parameters between four to six weeks and six months of age in these HIV-1 and CMV-exposed infants. The milk CMV DNA load remained only marginally negatively associated with the change in length-for-age in the adjusted analysis (−0.30; 95% CI: −0.86, 0.25).

As the milk CMV DNA and HIV-1 RNA loads were weakly positively associated in this cohort, we tested whether milk HIV-1 RNA virus load was a better predictor of infant growth at six months. None of the associations between HIV-1 RNA load and infant growth parameter *Z*-scores or change in growth parameter *Z*-scores at six months of age were significant (Table 3). However, there was a positive, though somewhat imprecise, association between infant weight-for-length at six months of age and HIV-1 RNA load in crude and adjusted analysis (Table 3). Thus, the correlations between milk CMV load and infant growth trends are stronger and more consistent than the associations between milk HIV-1 load and infant growth.

## 4. Discussion

HAART during pregnancy and breastfeeding is now standard practice for prevention of perinatal HIV-1 transmission [32]. However, this study was initiated prior to establishment of the impact of maternal HAART on postnatal HIV-1 transmission, allowing us to compare breast milk CMV shedding and CMV transmission in HAART-treated and untreated mothers. In our cohort, HAART initiated prior to or during pregnancy successfully prevented *in utero* or postpartum HIV-1 transmission, further demonstrating the success of maternal antiretroviral prophylaxis. Interestingly, women on HAART were more likely to have a low-birth-weight infant compared to untreated women (25% versus 2%, resp.), consistent with previous reports linking antiretrovirals during pregnancy with low birth weight [33–35]. However, this finding is in contrast to other recent data showing the lack of association between preterm birth and non-protease-inhibitor-containing maternal HAART regimens [36–38]. This finding could also be linked to the older age of women on therapy compared to those that were not. To determine the impact of maternal HAART on infant perinatal CMV exposure, we focused on the relationship between HAART and milk CMV shedding.

Studies have associated perinatal CMV infection with increased morbidity and mortality, not only in HIV-infected infants but also in HIV-exposed, uninfected infants. These groups may have impaired fetal and infant growth attributable to perinatal CMV infection [15, 39]. Despite the well-known risk of CMV and HIV-1 transmission via breast milk, breastfeeding is advocated as the primary source of infant nutrition in HIV-infected women in the developing world due to infeasible alternatives and high infant mortality associated with formula feeding [4]. Moreover, postnatal CMV transmission has been independently linked to breast milk CMV DNA load [21, 22]. Our study revealed a weak association in the magnitude of milk HIV-1 and CMV load, such that, for every HIV-1 log_10_ rise in HIV-1 load, there was a 0.39 log_10_ increase in CMV load. Gianella et al. similarly demonstrated a correlation between CMV and HIV-1 loads in other mucosal compartments, which is clinically significant as the presence of CMV is associated with HIV-1 disease progression and mortality [30, 40]. The correlation between CMV and HIV-1 shedding is independent of CD4+ T-cell count, plasma HIV-1 load, and other confounders [23, 41]. Despite these associations of mucosal HIV-1 and CMV shedding, our analysis revealed only minimal impact of HAART on breast milk CMV load, suggesting that expanded maternal use of antiretroviral therapy may have a limited impact on infant postnatal CMV exposure.

HIV-exposed, uninfected infants have a growth disadvantage compared to their unexposed counterparts [15], though the underlying pathophysiology is not understood. As congenital CMV infection is independently associated with *in utero* growth restriction, it is reasonable to consider whether postnatal CMV exposure or infection plays a role in the growth outcome of HIV-exposed, uninfected infants [42]. We found a reduced length-for-age *Z*-score (−0.53) and weight-for-age *Z*-score (−0.40) for each log_10_ increase in milk CMV load at 6 months of age in HIV-exposed, uninfected infants. However, the CMV milk DNA load remained only marginally negatively associated with the change in length-for-age *Z*-score between one and six months. Thus, the negative association of milk CMV DNA load with infant growth may only be significant in the early period of breastfeeding or only reflect the magnitude of the peripartum CMV exposure. Mixed feeding rates were high in this infant cohort before 6 months of life (14.9% prior to 5 months of age and 38.8% in the 6th month of life); thus, the growth rate over the first six months may have been impacted by this infant feeding pattern. Alternatively, high magnitude milk CMV shedding may be associated with maternal health status that is negatively impacting fetal and infant growth independently, as women that shed CMV at higher rates may be more immunocompromised and nutritionally deficient.

The limitations of this hypothesis-generating pilot study include a relatively small maternal sample size and lack of infant urine or saliva for confirmatory diagnosis of CMV congenital and postnatal CMV acquisition. Despite its small sample size, this study is unique in its enrollment of both HAART-treated and untreated mothers, as it enrolled prior to the establishment of maternal ARV treatment during breastfeeding as highly preventative against postnatal HIV transmission, elevating the importance of our analysis of CMV and HIV-1 load in milk and infant growth in this cohort. With these limitations in mind, we have demonstrated that maternal HAART does not have a large impact on breast milk CMV shedding, indicating that postnatal CMV exposure for HIV-exposed infants will continue at a similar level despite increasing maternal HIV-1 therapy/prophylaxis during breastfeeding. Moreover, the negative association between milk CMV load and postnatal growth in HIV-exposed infants in this small study is intriguing and should be further assessed in larger clinical studies, determining whether reduction of postnatal CMV exposure is important to improving the developmental outcome of HIV-exposed infants. Additional larger studies of the kinetics of maternal CMV shedding, postnatal transmission, and infant growth in the setting of maternal HAART would now be important given the current standard of care. Further understanding of the interaction between CMV and HIV-1 may establish methods to reduce the morbidity and growth impairment of HIV-exposed infants and maximize the benefits of breastfeeding in low-resource countries.

## Figures and Tables

**Table 1:** Clinical characteristics of HIV-infected lactating mothers and their infants, comparing HAART-treated and untreated women.

	Untreated *n* = 53	Treated *n* = 16	*P* value
Maternal characteristics			
Age, years	26 (23, 30)	33 (29, 36)	**0.0002**
Peripheral CD4 count (cells/*μ*L)^a^	382 (217, 494)	315 (257, 481)	0.74
Subclinical mastitis (milk K/Na > 1)^b^	15 (28%)	4 (25%)	0.80
Plasma HIV-1 RNA load (log⁡_10_⁡ copies/mL)^b^	4.0 (3.5, 4.8)	2.4 (2.4, 2.4)	<**0.0001**
Milk HIV-1 RNA load (log⁡_10_⁡ copies/mL)^b^	2.1 (2.1, 3.0)	2.1 (2.1, 2.1)	<**0.0001**
Milk CMV DNA load (log⁡_10_⁡ copies/mL)^b^	4.0 (3.2, 4.6)	4.0 (3.2, 5.3)	0.83
Infant characteristics			
*In utero* or peripartum HIV-1 acquisition	7 (13%)	0 (0%)	0.19
Postpartum HIV-1 acquisition	3 (6%)	0 (0%)	1.00
Congenital CMV acquisition	1 (2%)	2 (13%)	0.13
Low birth weight	1 (2%)	4 (25%)	**0.009**
Ballard score <37 weeks	12 (23%)	7 (44%)	0.12

Results reported as median (IQR: interquartile range) or *N* (%). Two-sided *P* values for maternal characteristics derived from Wilcoxon two-sample test, normal approximation, or two-sided *P* values for infant characteristics from Fisher's exact test. Significant *P* values (*P* < 0.05) are bolded.

^a^Measured in the 3rd trimester of pregnancy.

^b^Measured at four to six weeks postpartum.

**Table 2:** Effect of maternal HAART on milk CMV DNA load at four to six weeks postpartum, measured as difference in milk log⁡_10_⁡  CMV DNA load associated with HAART use.

	Crude	Adjusted^a^
Both breasts	0.08 (−0.59, 0.74)	−0.33 (−0.95, 0.30)
Left breast	0.16 (−0.50, 0.82)	−0.21 (−0.90, 0.49)
Right breast	−0.00 (−0.66, 0.65)	−0.50 (−1.18, 0.17)

^a^Results reported as difference in milk log⁡_10_⁡  CMV DNA associated with HAART use, controlling for infant gestational age and birth weight and maternal age, mastitis, and CD4 count.

**Table 3:** Association between breast milk log⁡_10_⁡  CMV DNA and HIV-1 RNA load and growth of breastfed HIV-exposed, uninfected infants.

Outcome measure (*n* = 55)	CMV DNA load associations		HIV-1 RNA load associations	
	Crude	Adjusted^c^	Crude	Adjusted
Six-month *Z*-score^a^	*Z* (95% CI)	*Z* (95% CI)	*Z* (95% CI)	*Z* (95% CI)

Weight for length	−0.05 (−0.40, 0.29)	−0.03 (−0.36, 0.30)	0.30 (−0.03, 0.63)	0.40 (−0.04, 0.83)
Length for age	−0.48 (−0.99, −0.08)	−0.52 (−0.93, −0.10)	−0.17 (−0.59, 0.26)	−0.35 (−0.92, 0.21)
Weight for age	−0.40 (−0.67, −0.12)	−0.39 (−0.66, −0.13)	0.13 (−0.17, 0.42)	0.11 (−0.26, 0.47)

Change in *Z*-score^b^	Δ*Z* (95% CI)	Δ*Z* (95% CI)	Δ*Z* (95% CI)	Δ*Z* (95% CI)

Weight for length	0.63 (−0.05, 1.30)	0.72 (0.04, 1.40)	0.22 (−0.47, 0.91)	−0.62 (−1.50, 0.27)
Length for age	−0.04 (−0.56, 0.48)	−0.29 (−0.84, 0.26)	−0.39 (−0.93, 0.15)	0.20 (−0.55, 0.96)
Weight for age	0.20 (−0.03, 0.43)	0.16 (−0.05, 0.38)	−0.01 (−0.25, 0.23)	−0.04 (−0.34, 0.26)

^a^Numbers in the table represent difference in *Z*-score at six months of age associated with a one-log_10_ increase in average breast milk CMV load.

^b^Numbers in the table represent difference in differences: the change in delta-*Z*-score between six months of age and four to six weeks of age associated with a one-log_10_ increase in average breast milk CMV load.

^c^Adjusted analysis accounts for infant birth weight and gestational age and maternal HAART use, age, and CD4 count.
